# Investigation of Electric Field Tunable Optical and Electrical Characteristics of Zigzag and Armchair Graphene Nanoribbons: An Ab Initio Approach

**DOI:** 10.3390/nano14171446

**Published:** 2024-09-04

**Authors:** Recep Emir, Cagatay Tuncsiper, Dilek Surekci Yamacli, Serhan Yamacli, Sezai Alper Tekin

**Affiliations:** 1 Department of Electrical-Electronics Engineering, Erciyes University, 38010 Kayseri, Turkey; 2 Centrade Fulfillment Services Ltd., 35010 Izmir, Turkey; tuncsipercgty2@gmail.com; 3 Department of Economics, Izmir Democracy University, 35140 Izmir, Turkey; dilek.surekciyamacli@idu.edu.tr; 4 Department of Biomedical Engineering, Izmir Democracy University, 35140 Izmir, Turkey; serhan.yamacli@idu.edu.tr; 5 Department of Industrial Design Engineering, Erciyes University, 38010 Kayseri, Turkey; satekin@erciyes.edu.tr

**Keywords:** graphene nanoribbons, DFT, optical properties, Kubo–Greenwood formalism, Landauer approach

## Abstract

Graphene nanoribbons (GNRs), categorized into zigzag and armchair types, hold significant promise in electronics due to their unique properties. In this study, optical properties of zigzag and armchair GNRs are investigated using density functional theory (DFT) in conjunction with Kubo–Greenwood formalism. Our findings reveal that optical characteristics of both GNR types can be extensively modulated through the application of a transverse electric field, e.g., the refractive index of the a zigzag GNR is shown to vary in the range of n = 0.3 and n = 9.9 for the transverse electric field values between 0 V/Å and 10 V/Å. Additionally, electrical transmission spectra and the electrical conductivities of the GNRs are studied using DFT combined with non-equilibrium Green’s function formalism, again uncovering a strong dependence on the transverse electric field. For example, the conductance of the armchair GNR is shown to vary in the range of G = 6 μA/V and G = 201 μA/V by the transverse electric field. These results demonstrate the potential of GNRs for use in electronically controlled optoelectronic devices, promising a broad range of applications in advanced electronic systems.

## 1. Introduction

Graphene nanoribbons (GNRs) have emerged as promising candidates for a variety of applications in the field of nanoelectronics and optoelectronics due to their unique electronic and optical properties. GNRs can be categorized into two main types based on their edge configurations, i.e., zigzag and armchair GNRs. These configurations have distinct characteristics making them highly versatile materials for developing next-generation electronic devices.

There are vast numbers of theoretical and practical studies in the literature regarding the optical properties of graphene and GNRs. In one of the earliest works, the optical conductivity of graphene is derived and it is shown that a gate voltage can be utilized to change the longitudinal optical conductivity [1]. In another study, the optical reflectance behaviors of single and multi-layer graphene are obtained as a function of the temperature and the carrier density [2]. The optical characteristics of graphene are studied by Falkovsky in another work, where the dynamic conductance of monolayer and multilayer graphene are derived dependent on the temperature and the chemical potential [3]. The optical reflectivity and transmission of single-layer graphene on SiO_2_ substrate are measured by Mak et al., where it is observed that the optical absorbance of single-layer graphene is almost flat for photon energies above 0.5 eV [4]. In another experimental paper, Nair et al. exposed that the visual transparency of graphene is dependent on the fine structure constant [5]. The relation between the optical transparency of graphene and the fine structure constant is theoretically verified also by Sheehy and Schmalian [6]. In another work, a non-interacting tight-binding model is utilized by Stauber et al. beyond the Dirac-cone approximation, where it is shown that the optical conductivity of graphene is found to in the order of (*ħω/t*)^2^ [7]. The gate voltage dependency of the optical behaviors of single and bilayer graphene are experimentally studied using infrared spectroscopy by Wang et al. and it is shown that the inter-band transitions of these materials can be varied using a gate voltage [8]. The optical constants of few and thick graphene layers are computed within the Fresnel coefficient calculation procedure by Bruna and Borini, where it is demonstrated that the complex refractive index can be used to estimate the optical behavior in the visible range [9]. In another experimental work, the nonlinear optical behavior of single and few-layer graphene are measured employing four-wave mixing method and it is observed that graphene has strongly nonlinear optical response in the near-infrared region [10]. The optical reflection and transmission characteristics of monolayer to several hundred layers of graphene are experimentally studied by Skulason et al. and it is shown that the reflection and transmission of graphene in the visible layers are strongly dependent on the real part of the optical conductance and weakly dependent on the imaginary part of the optical conductance [11]. The optical transmission and reflection properties of large-scale graphene produced by chemical vapor decomposition (CVD) is measured in another work by Lee et al., where it is observed that the single layer optical response is consistent over the entire energy range of 4 meV–6.2 eV [12]. The optical behavior of graphene at a strong terahertz field is theoretically investigated by Zhou and Wu using Floquet theory and it is shown that the optical conductivity of graphene shows a multiple step-like structure dependent on the modulated optical transitions [13]. The optical response of graphene is extensively studied by Mak et al., where they have demonstrated that the optical absorption of graphene is dominated by intra-band and inter-band transitions for far-infrared and mid-infrared to ultraviolet spectra, respectively [14]. The transient optical conductivity of graphene is experimentally investigated using femtosecond time resolved spectroscopy by Malard et al., where it is observed that the optical conductivity of graphene is influenced by the absorption from intra-band transitions of carriers and the bleaching of inter-band transitions [15]. A perturbative computational analysis is performed by Cheng et al. on the third order optical conductivities of graphene, where it is shown that strong optical nonlinearity can be achieved by varying the chemical potential [16]. Density functional theory (DFT) calculations are utilized to study the optical properties of pure and doped graphene by Rani et al., where they have shown that DFT computation results regarding the optical response are in agreement with the experimental values [17]. In another work, the optical transmittance measurements combined with tight-binding simulations performed by Zhu et al. demonstrates that the optical transmission of graphene films is only determined by the number of graphene layers [18]. The nonlinear optical characteristics of multilayer graphene are experimentally studied by Demetriou et al. using z-scan method and they have observed negative nonlinear refraction in graphene [19]. Similarly, a negative nonlinear refractive index is observed for monolayer graphene by Dremetsika et al. using the ultrafast optical Kerr effect technique [20]. In another work, the optical characteristics of CVD-produced graphene is obtained in the range of 0.7 eV–9.0 eV employing spectroscopic ellipsometry, where it is shown that the absorbance of graphene is almost constant in the visible range [21]. The semiclassical theory of the optical properties of graphene is studied by Semnani et al. to obtain the linear and nonlinear optical characteristics of graphene and it is demonstrated that the nonlinear optical response of graphene is a result of pure inter-band and intra-band transitions together with their interplay [22]. Baudsich et al. have investigated the optical properties of charge carriers in graphene and the ultrafast optical field dependent behavior of Dirac fermions were exposed [23]. In another study, the effects of doping on the electrical and optical properties of graphene are investigated and it is exposed that doping strongly affects the optical response of graphene due to the variations in the increment of excitation channels [24]. The peculiar optical response of graphene is also utilized to produce practical devices in the literature, e.g., the absorption peaks of graphene at 2.58 THz and 6.07 THz are used for prototyping a dual-band terahertz sensor employing graphene [25]. 

The optical characteristics of armchair GNRs are studied by Yang et al. utilizing first-principles computations, where they have shown that excitonic effects are dominant in determining the optical response of armchair GNRs due to the reduced dimensionality [26]. In another work, the effects of dimensionality reduction and longitudinal polarized electromagnetic field on the optical characteristics of armchair GNRs are studied by Liao et al. and they have shown that the real conductance and the dielectric function can be modulated by infrared to ultraviolet radiations [27]. Wright et al. have studied the optical response of bilayer GNRs and have found that a subclass of bilayer GNRs have optical conductivities on the order of twice the conductance quantum in the terahertz and far infrared spectra [28]. In another work, the optical response of GNRs are investigated using the Peierls tight-binding model combined with gradient approximation, where it is shown that the absorption spectra of GNRs can be tuned using a spatially modulated magnetic field [29]. The optical absorption spectra of armchair GNRs are studied using first-principles computations in another work and it is demonstrated that the optical absorption of armchair GNRs can be tuned via the shape and the width of the ribbon [30]. Similarly, the excitonic binding energies and the optical properties of armchair GNRs are studied using the tight-binding model in another work where it is shown that the peaks in the absorption spectrum are dependent on the energy bandgap [31]. The optical response of both zigzag and armchair GNRs are investigated employing tight-binding model by Sasaki et al. and it is shown that intra-band indirect transition occurs in cases where the light polarization is perpendicular to the GNR edges [32]. Li et al. have shown that the optical properties of curved GNRs can be modulated using an external electric field, which is caused by the electron transfers among atoms inducing the deformation of the wave function [33]. Similarly, Alaei and Sheiki have investigated the electrical and optical effects of the external transverse and longitudinal electric fields on GNRs using tight-binding model, where they have argued that external electric fields cause changes in the energy dispersions and bandgap leading to the tunability of the electrical and optical behavior of GNRs [34]. On the other hand, Denk et al. have studied the optical absorption spectra of ultra narrow armchair GNRs using both first-principles methods and experimental measurements and they have shown that the optical absorption peaks of the N = 7 armchair GNR are located at the energy values of 2.1 eV, 2.3 eV and 4.2 eV, in agreement with the first-principles computations [35]. In another work, it is experimentally demonstrated that functionalized GNRs can be used to transit radio frequency power density up to 2 × 10^5^ W/m^2^ [36]. The optical characteristics of zigzag and armchair graphene nanoribbons, which have triple carbon-carbon bonds, are studied by Asadpour et al., where it is exposed that the optical bandgap of graphene nanoribbons decrease by the ribbon width [37]. The optical properties of chiral GNRs are investigated by Berahman et al. in another work where it is observed that the selection rules established for zigzag and armchair GNRs are not valid for chiral GNRs due to breaking the symmetry, and it is also shown that the absorption peaks of chiral GNRs are located at around 1000 nm [38]. The electrical and optical characteristics of armchair GNRs are obtained by Hassan et al. using theoretical analysis and it is concluded that armchair GNR has high transparency for E_F_ > k_B_T, where E_F_ is Fermi energy, k_B_ is Boltzmann constant and T is absolute temperature [39]. The plasmonic response of GNR arrays are analytically investigated by Velizhanin and it is shown that the size quantization condition for graphene plasmons has purely geometric conditions [40]. In an experimental work, nine-atoms-wide armchair GNRs are implemented employing a CVD method and then the optical conductivity of these GNRs is measured using time-resolved terahertz spectroscopy, where the photoconductivity value is determined as 350 cm^2^V**^−^**^1^s**^−^**^1^ [41]. On the other hand, Zhou et al. have investigated the fundamental and second harmonic waves in GNR arrays using a finite-difference time-domain method combined with coupled mode theory and it is exposed that graphene plasmonic waves are induced by the enhancement of the local field [42]. In another study, the tight-binding model in conjunction with Green’s function approach is utilized to study the effects of the ribbon width, chemical potential and an external magnetic field on the optical absorption of armchair GNRs and it is demonstrated that the application of the external magnetic field increases the optical conductivity up to a peak value and the further increment of the external magnetic field then leads to the decrement of the optical conductivity [43]. Hasani and Chegel investigated the effects of the external electric field on the electrical and optical behavior of armchair GNRs using first-principles computations, where it is shown that the external electric field decreases the energy bandgap leading to the changes the peaks of the optical absorption spectrum and an increment in the dielectric constant of armchair GNRs [44]. Similarly, Jabbar and Kadhim studied the linear and nonlinear optical behavior of GNRs employing time-dependent DFT method and they concluded that the optical bandgap of the GNRs can be changed via doping [45]. In another work, Uryu has studied the optical characteristics of zigzag and armchair GNRs using effective mass approximation for intrinsic and doped cases and it is shown that the dynamical conductivity of GNRs is affected by the doping concentration as a result of the changes in the energy level variations [46]. Klimenko et al. have experimentally investigated the plasmons in GNR arrays and have explained the measurement results using canonical non-interacting theory, concluding that plasmon–plasmon and plasmon–radiative interactions affect plasmonic energy redshifts [47]. Sheridan et al. have also performed experimental studies regarding the optical characteristics of GNRs using the LaAlO_3_/SrTiO_3_ interface, where it is demonstrated that the optical response of GNR nanoclusters have strong gate-tunable properties [48]. Zhang et al. have employed first-principles computations regarding the spin current generation in zigzag GNRs and they have shown that the spin current can be generated via the photovoltaic effect in zigzag GNRs [49]. Ge and Fisher have also used first-principles calculations to investigate the optical properties of single-layer and bilayer GNRs and they have demonstrated that the optical characteristics of GNRs depend on the ribbon width, number of layers and the edge alignment [50]. Jiang et al. have experimentally investigated the optical characteristics of GNRs laid on a partially insulated surface and they have observed that localized dark excitons are associated with the topological end states in GNRs [51]. On the other hand, Nguyen et al. have studied armchair and bilayer GNRs employing tight-binding computations and have demonstrated that optical absorption spectra of GNRs can be changed by the ribbon width or an external electric field [52]. Zhang et al. have utilized DFT computations for exploring the effects of the stress on the optical properties of zigzag and armchair GNRs and they have concluded that the absorption spectra of GNRs have redshift trend as the stress increases [53]. Similarly, Liu et al. have studied the effects of the stress on the optical characteristics of graphdiyne nanoribbons utilizing DFT simulations and they have observed that zigzag graphdiyne nanoribbons have wider range plasmon effect compared to their armchair counterparts [54]. In another work, Rezania and Kakavandi employed tight-binding computations for extracting the optical absorption characteristics of doped armchair GNRs where they have found that the optical absorption have increasing trend as the doping concentration increases [55]. In addition, the dynamic conductivity of graphene, plasmon polaritons and graphene-based meta-devices are extensively reviewed by Zeng et al. [56]. On the other hand, Jiang et al. have presented a graphene layer based in-fiber device which can be used for photoelectric conversion and electrically-induced thermo-optic effects implying the practical optical applications of a graphene layer [57]. Similarly, Li et al. have proposed a single-layer graphene based tunable bandwidth absorber for use in biomedical, space science and communication applications. also demonstrating the practical optical potentials of graphene [58].

The electronic transport properties of zigzag and armchair GNRs are also investigated in detail in the literature. In one of the earlier studies, Wakabayashi studied the electrical transport properties of zigzag GNRs using Landauer–Büttiker formalism, where it is shown that zigzag GNRs have single conducting channels of edge states [59]. Munoz-Rojas et al. have also investigated the transport properties of zigzag GNRs and they have concluded that low-energy transport characteristics of zigzag GNRs are robust to edge vacancies [60]. Peres et al. also utilized Landauer–Büttiker formalism for the extraction of the electrical characteristics of armchair and zigzag GNRs and they discovered that the conductance quantization of GNRs depend on their edge configurations [61]. In another work, the electron-phonon scattering effects in zigzag GNRs are studied by Gunlycke et al., where they have estimated the mean free path of electrons in zigzag GNRs to be approximately 70 μm [62]. Li et al. have also investigated the electrical transport properties of zigzag GNRs from the symmetry viewpoint using first-principles computations and they have shown that, while asymmetrical zigzag GNRs have linear current-voltage characteristics, symmetrical zigzag GNRs have a large bandgap due to the coupling between π and π* sub-bands [63]. Kimouche et al. performed experimental studies regarding the electronic transport properties of 5-atoms-wide ultra-narrow armchair GNRs and concluded that these armchair GNRs had a bandgap of around 100 meV and edge defects have limited effects on their electronic transport properties [64]. In a recent work, Lopez-Urias et al. have investigated the electrical characteristics of armchair GNRs and have argued that armchair GNRs with n = m = 3p + 2, where p is an integer, display metallic behavior similar to zigzag GNRs [65]. These studies demonstrate that electrical and optical characteristics are explored in detail in the literature owing to the fact that GNRs are promising candidates for a wide array of optoelectronic applications.

The recent advances in DFT have enabled detailed investigations into the properties of nanomaterials such as GNRs, providing deep insights into their behavior under different conditions. This study aims to explore tunable optical and electrical characteristics of GNRs in detail utilizing DFT combined with Kubo–Greenwood formalism for optical properties and non-equilibrium Green’s function (NEGF) formalism for electrical transmission characteristics. Our findings demonstrate the profound impact of transverse electric fields on the permittivity, refractive indices, reflectivity and conductance of zigzag and armchair GNRs, highlighting their potential for innovative applications in advanced optoelectronic systems.

## 2. Materials and Methods

**Figure 1:**
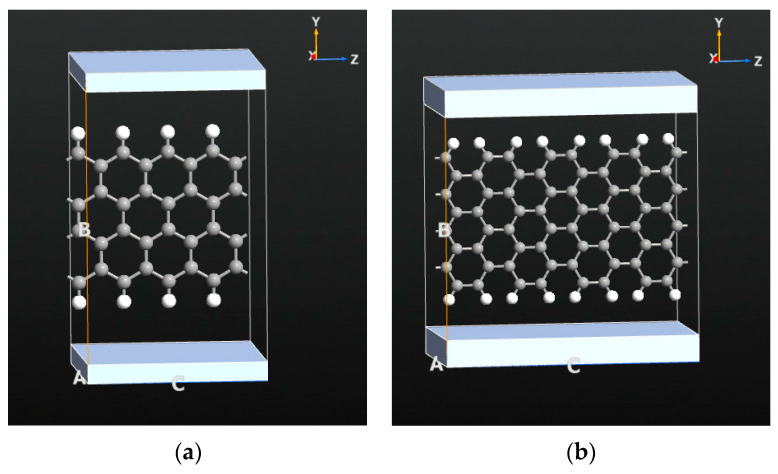
The two main types of GNR considered. (**a**) The 8-atoms wide zigzag GNR; (**b**) equivalent 8-atoms wide armchair GNR.

A zigzag and an armchair GNR are studied in this work, considering that these are the two main classes of GNRs. Both GNRs are 8-atoms-wide structures, as shown in Figure 1. The *z*-axis repetitions of the GNRs are taken as 4 considering the accuracy and the simulation cost. On the other hand, as can be seen from Figure 1, there are also two electrodes at the top and the bottom of the unit cell for the application of the transverse electric field along the *y*-axis. The geometric structures of the GNRs are relaxed before the actual density functional computations regarding the effect of the transverse electric field on the optical and the electrical characteristics of the GNRs. The geometric optimizations of the GNRs are performed using the density functional theory (DFT) with the generalized gradient approximation for the exchange-correlation functional [GGA], double-zeta polarized basis sets, FHI pseudopotentials and 200 Ry of cut-off energy for the real-grid mesh [66,67]. The limited memory Broyden–Fletcher–Goldfarb-Shanno (LBFGS) algorithm is utilized with a force tolerance of 0.01 eV/Å [68]. 

The carbon–carbon and carbon–hydrogen bond distances are obtained as 1.42 Å and 1.09 Å, respectively, which are compliant with the values existing in the literature [69,70,71,72,73]. After the geometric optimization step, the actual DFT computations are performed for obtaining the optical and electrical properties of the considered GNRs and their variation with the applied transverse electric fields. The thickness, length and width of the studied zigzag GNR sample are 3.40 Å, 8.64 Å and 9.28 Å, respectively. Similarly, the armchair GNR sample has 3.40 Å, 15.63 Å and 10.51 Å of thickness, length and width, respectively. The electric fields are introduced to the structures via the electrodes shown at the top and the bottom of the GNRs in Figure 1.

The DFT computations for the optical and electrical behavior are performed using Quantumwise ATK with the following parameters: GGA exchange-correlation functional, 250 Ry of mesh density cut-off energy, double-zeta polarized basis sets and multigrid Poisson solver with Neumann type boundary conditions for the top and bottom of the unit cells [74,75]. Kubo–Greenwood formalism is utilized in Quantumwise ATK for obtaining the susceptibility tensor (χ) dependent on the angular frequency (*ω*), as shown in Equation (1).

(1) $$\chi_{ab}\left(\omega \right)=\frac{q^{2}}{\hbar m^{*}\Omega }\sum_{nmk}\frac{f_{mk}-f_{nk}}{\left(\omega_{nm}-\omega -i\Gamma /\hbar \right)\omega_{nm}^{2}}\pi_{nm}^{a}\pi_{mn}^{b}$$

where *q* is the elementary charge, *ħ* is the reduced Planck’s constant, m* is the electron effective mass, Ω is the unit cell volume, *f* is the Fermi–Dirac probability distribution function, Γ is the energy broadening and *π* is the momentum operator between states *n* and *m* [76,77,78,79]. Complex relative permittivity, refractive index and reflectivity can be obtained from the susceptibility function using Equations (2)–(4), respectively.

(2) $$\varepsilon_{r}\left(\omega \right)=\varepsilon_{r}\left(\omega \right)+i\varepsilon_{i}\left(\omega \right)=\left[1+\chi \left(\omega \right)\right]$$

(3) $$n\left(\omega \right)=\sqrt{\frac{\sqrt{{\varepsilon_{r}}^{2}+{\varepsilon_{i}}^{2}}+\varepsilon_{r}}{2}}$$

(4) $$R\left(\omega \right)=\frac{{\left(1-\sqrt{\frac{\sqrt{{\varepsilon_{r}}^{2}+{\varepsilon_{i}}^{2}}+\varepsilon_{r}}{2}}\right)}^{2}+\frac{\sqrt{{\varepsilon_{r}}^{2}+{\varepsilon_{i}}^{2}}-\varepsilon_{r}}{2}}{{\left(1+\sqrt{\frac{\sqrt{{\varepsilon_{r}}^{2}+{\varepsilon_{i}}^{2}}+\varepsilon_{r}}{2}}\right)}^{2}+\frac{\sqrt{{\varepsilon_{r}}^{2}+{\varepsilon_{i}}^{2}}-\varepsilon_{r}}{2}}$$

where *ε_r_* and *ε_i_* are the real and imaginary parts of the permittivity, *ε*_0_ is the vacuum permittivity and *ω* is the angular frequency [77,80,81]. Therefore, complex relative permittivity, optical conductivity, refractive index and reflectivity can be computed after the susceptibility is obtained via the Kubo–Greenwood formula in conjunction with the DFT simulations.

The electrical behavior of nanoscale structures can be obtained using DFT combined with the non-equilibrium Green’s function (NEGF) formalism [82,83,84]. The electrical transmission coefficients at energy points *E* can be obtained using the retarded Green’s function, as shown in Equation (5).

(5) $$T\left(E\right)=G\left(E\right)\Gamma^{L}\left(E\right)G^{M}\left(E\right)\Gamma^{R}\left(E\right)$$

where *G*(*E*) is the retarded Green’s function and Γ is the energy broadening function, superscripts denoting the left electrode, center and the right electrodes [77]. In addition, the conductance value can also be computed using the Landauer formula employing the transmission coefficients *T_n_(E),* as in Equation (6) [82,85,86,87].

(6) $$G=\frac{2q^{2}}{h}\sum_{n}T_{n}\left(E\right)$$

Therefore, both optical and electrical characteristics of the considered zigzag and armchair GNRs can be computed using the DFT approach in conjunction with the Kubo–Greenwood and NEGF formalism, as performed in this study.

The transverse electric field is applied using the electrodes shown at the top and bottom sides of the GNR structures in Figure 1. The distance between the electrodes is set as 15 Å and the electric field between these electrodes, which is in the transverse direction of the GNRs, sweeps between 0 V/Å and 10 V/Å at 11 evenly spaced values. These electric field values are selected to be realistic values that can be achieved practically. The optical response and electrical transmission characteristics of the GNRs are computed for each electric field value using the DFT computations. The optical and electrical behaviors of both zigzag and armchair GNRs show strong dependence on the applied electric field, as explained in detail in the next section. 

## 3. Results and Discussion

### 3.1. Electric-Field-Dependent Optical Characteristics of the GNR Samples

**Figure 2:**
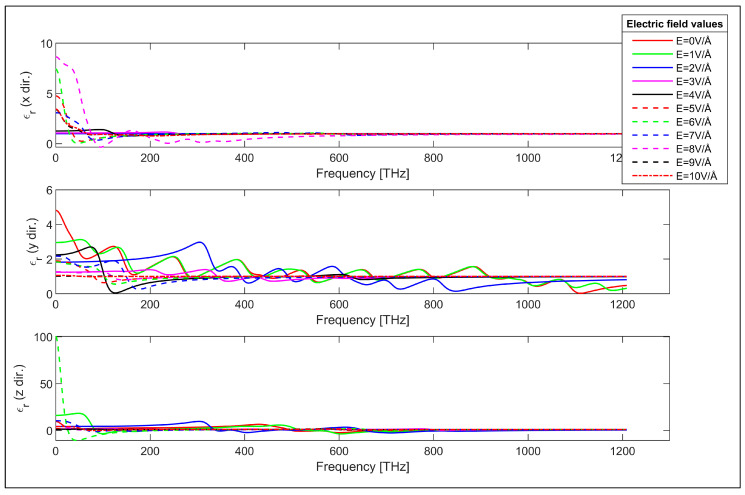
Real part of the permittivity of the zigzag GNR sample in x, y and z directions.

**Figure 3:**
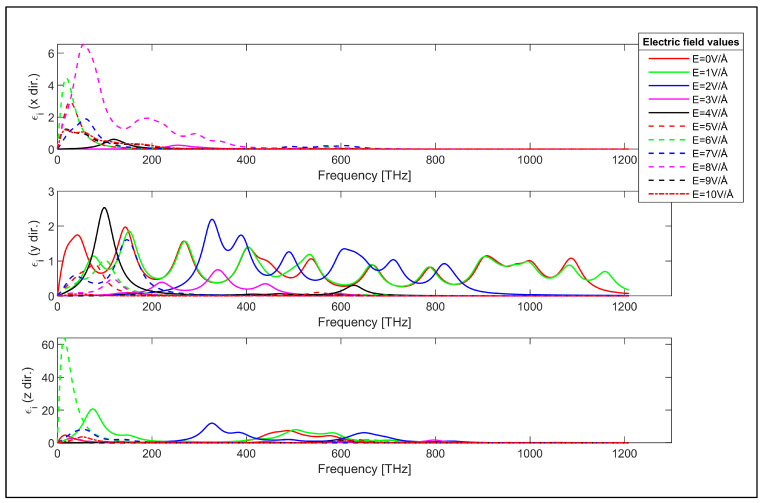
Imaginary part of the permittivity of the zigzag GNR sample in x, y and z directions.

The optical properties of the investigated zigzag and armchair GNRs are obtained employing the DFT in conjunction with Kubo–Greenwood formalism as the first step. The frequency dependent real and imaginary components of the relative permittivity of the zigzag GNR sample are plotted in Figure 2 and Figure 3 and for various values of the applied transverse electric field. It can be observed from Figure 2 and Figure 3 that both the real and imaginary components of the relative permittivity of the zigzag GNR sample can be varied using the applied transverse electric field. The changes in the permittivity components occur in all x, y and z directions, but the variation is greater in the y direction due to the direction of the transverse electric field. Moreover, the permittivity components can be changed in infrared, visible and ultraviolet regions, indicating the possibility of the utilization of the zigzag GNR for electric field controlled optical components for all these spectra.

The characteristics of Figure 2 show that the real part of the permittivity in the x direction takes positive values across the considered frequency range while they can have negative values in y and z directions. Furthermore, the real part of the permittivity in the z direction has much higher values compared to the real permittivity in x and y directions. This can be explained considering the susceptibility tensor given in Equation (1). The momentum matrix elements take higher values in the z direction due to the periodicity. This is also observed in the last plot shown in Figure 3 for the imaginary values of the permittivity in the periodic direction. It is worth noting that the transverse electric field’s controllable real and imaginary parts of the frequency dependent permittivity characteristics in Figure 2 and Figure 3 demonstrate a peculiar behavior that can be exploited for the design of electronically controllable optical components utilizing zigzag GNRs.

**Figure 4:**
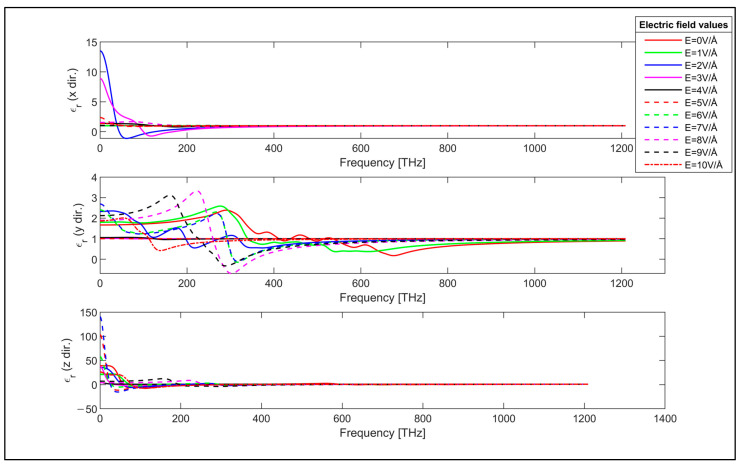
Real part of the permittivity of the armchair GNR sample in x, y and z directions.

**Figure 5:**
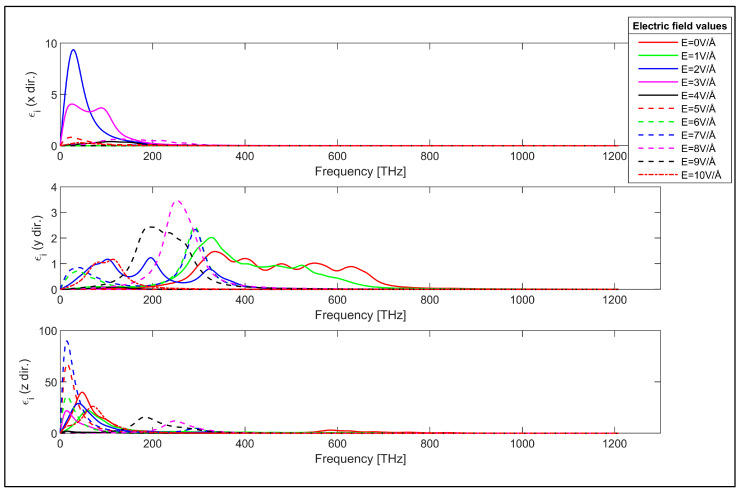
Imaginary part of the permittivity of the armchair GNR sample in x, y and z directions.

Similarly, the real and imaginary parts of the relative permittivity of the considered armchair GNR sample are also plotted and analyzed, as in Figure 4 and Figure 5, respectively. The relative permittivity values of the armchair GNR sample show that the transverse electric field can be used to tune the optical characteristics of the armchair GNR, similar to the zigzag GNR case.

The x-direction values of the real and imaginary parts of the permittivity of the armchair GNR sample are negatively modulated by the strength of the applied transverse electric field, as observed from the uppermost plots of Figure 4 and Figure 5. In contrast, the real and imaginary components of the permittivity of the zigzag GNR sample are positively affected by the applied electric field, as shown in the topmost plots of Figure 2 and Figure 3. In addition, the y-direction values of the real and imaginary components of the permittivity values display stronger dependency on the transverse electric field compared to those of the x and z-direction values for both zigzag and armchair GNR samples. This is expected, since the transverse electric field has a higher effect on the band-structure and the momentum matrix elements in the y-direction for both zigzag and armchair GNRs. Furthermore, real and imaginary components of the permittivity values of the armchair GNR have higher values in the z-direction, which is the periodic direction, compared to the x and y-direction values as in the zigzag GNR case. The z-direction real and imaginary values of the permittivity of the armchair GNR sample takes higher values than those of the zigzag GNR sample due to the values of the momentum matrix elements.

**Figure 6:**
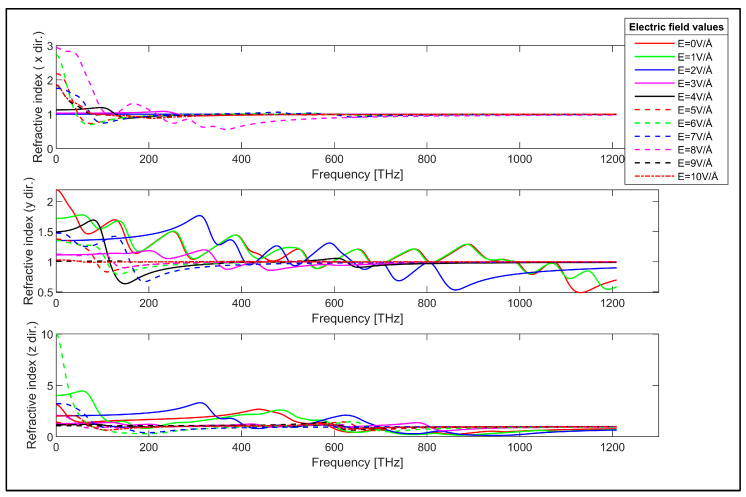
Refractive index of the zigzag GNR sample in x, y and z directions.

The refractive indices of the zigzag and armchair GNR samples are obtained using the susceptibility tensor and Equation (3). The x, y and z-direction refractive index of the zigzag GNR sample is plotted in Figure 6.

The refractive index in the x-direction of the zigzag GNR sample is close to unity for lower values of the applied electric field and the shape of this refractive index can be changed from unity by the applied electric field. On the other hand, the refractive index of the zigzag GNR sample in the y-direction shows a strong frequency dependence, as can be observed from the middle plot in Figure 6. However, this refractive index can be decreased and stabilized by the application of the transverse electric field. The maximum values of the x and y-direction refraction indices of the zigzag GNR sample are close to each other. The z-direction refractive index of this sample can also be decreased by the applied electric field, as can be seen from the bottom plot in Figure 6. Moreover, the maximum value of the refractive index can reach n = 9.92 for low frequencies in the z-direction for the applied electric field value of E = 6 V/Å. It is worth noting that the refractive index of the zigzag GNR sample can be controlled by the applied transverse electric field in x, y and z-directions and for infrared, visible and ultraviolet spectra.

**Figure 7:**
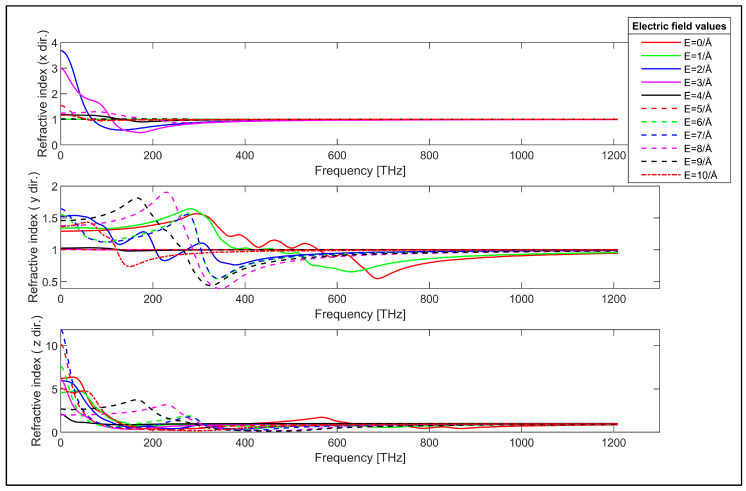
Refractive index of the armchair GNR sample in x, y and z directions.

The refractive indices of the armchair GNR sample are displayed for x, y and z-directions in Figure 7. The uppermost plot in Figure 7 shows that the x-direction refractive index of this GNR sample can be controlled below about 400 THz by the applied transverse electric field. Moreover, the x-direction refractive index is close to unity except for the applied electric field values of E = 2 V/Å and E = 3 V/Å, which cause the x-direction refractive index to reach n = 2.96 and n = 3.67 for the low frequency range. The y-direction refractive index is shown in the middle plot in Figure 7. It can be observed from this plot that the y-direction refractive index shows strong transverse electric field dependence as expected. The z-direction refractive index can reach n = 11.74 for the applied transverse electric field of E = 7 V/Å. Moreover, the z-direction refractive index takes values in the range of n = 1.4 and n = 0.3 for various values of the applied electric field. It is worth noting that the refractive index of the armchair GNR sample can be controlled by the applied transverse electric field in y-direction for infrared, visible and ultraviolet spectra, while the refractive index in x and z-directions have low dependency on the applied electric field.

**Figure 8:**
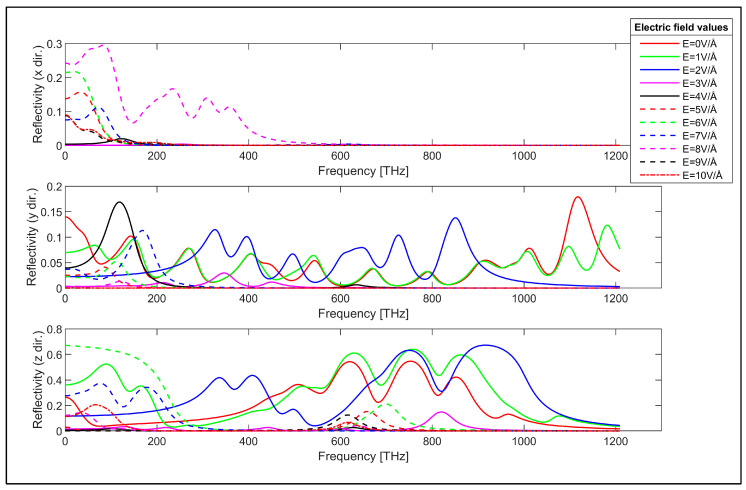
Reflectivity of the zigzag GNR sample in x, y and z directions.

**Figure 9:**
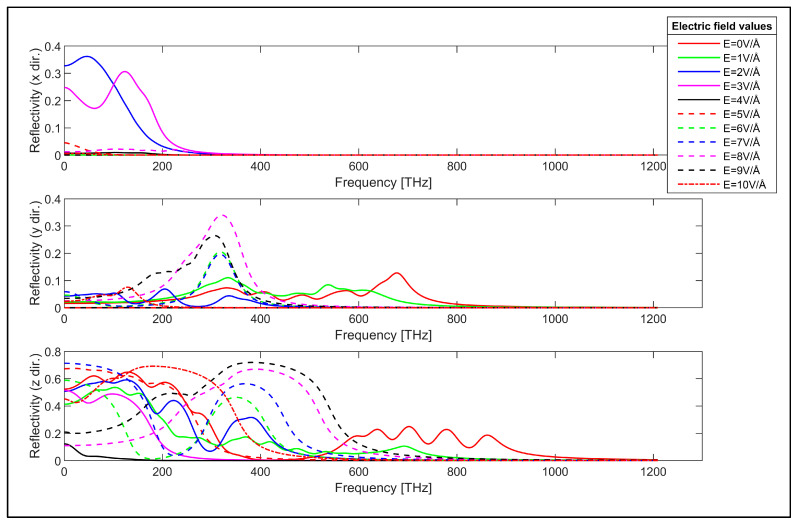
Reflectivity of the armchair GNR sample in x, y and z directions.

The reflectivity of the considered zigzag and armchair GNR samples is obtained using Equation (4) as the next step. The reflectivity of the zigzag GNR sample for the x, y and z-directions are plotted in Figure 8. Reflectivity is an important parameter that enables interpretation of the practical reflection and transmission of the incident electromagnetic energy. The reflectivity of the zigzag GNR sample in the x-direction is given by the top plot in Figure 8. According to this plot, the reflectivity of the zigzag GNR sample is close to zero for the absence of the transverse electric field. However, the application of the electric field can be used to modulate the reflectivity in the x-direction for infrared and visible spectra. On the other hand, the reflectivity in the y-direction shows strong dependence on the applied transverse electric field for the infrared, visible and ultraviolet spectra, as can be observed from the middle plot in Figure 8. This argument is also valid for the reflectivity in the z-direction, as can be seen from the bottom plot in Figure 8. The reflectivity of the armchair GNR sample in x, y and z-directions are given in Figure 9.

The reflectivity of the armchair GNR sample in the x-direction is also close to zero for the absence of the transverse electric field, which is similar to the zigzag GNR case, as can be seen from the top plot in Figure 9. The x-direction reflectivity of the armchair GNR sample can be modulated by the transverse electric field for infrared and lower frequency range, as can be observed from this plot. The y-direction reflectivity of the armchair GNR sample is plotted in the middle plot in Figure 9. The reflectivity has a finite value for the absence of the transverse electric field and it can be varied by the application of the transverse electric field. The reflectivity of the armchair GNR sample increases up to 0.36 in the infrared spectrum for higher values of the applied electric field. The reflectivity in the y-direction again shows strong dependence on the value of the transverse electric field as expected. The z-direction reflectivity of the armchair GNR sample is shown in the bottom plot in Figure 9. The reflectivity again has infinite value in the absence of the transverse electric field. However, the application of the transverse electric field strongly modulates the reflectivity for infrared, visible and ultraviolet spectra. For example, the z-direction reflectivity can take values up to 0.71 in the visible spectrum for the transverse electric field having the value of E = 9 V/Å. The transverse electric field modulation of the reflectivity of the zigzag and armchair GNR samples indicates the possible utilization of GNRs for electronically controllable optical devices. The measured refractive index values for graphene in the absence of transverse electric field values are reported to be in the range of n = 0.5 to n = 3.4 in the literature [88,89,90]. The refractive index values for the absence of electric field (E = 0V/Å) in our study are consistent with the reported experimental values as observed from Figure 6 and Figure 7 for the zigzag and armchair GNR samples. 

### 3.2. Electric-Field-Dependent Electrical Transmission Characteristics of the GNR Samples

**Figure 10:**
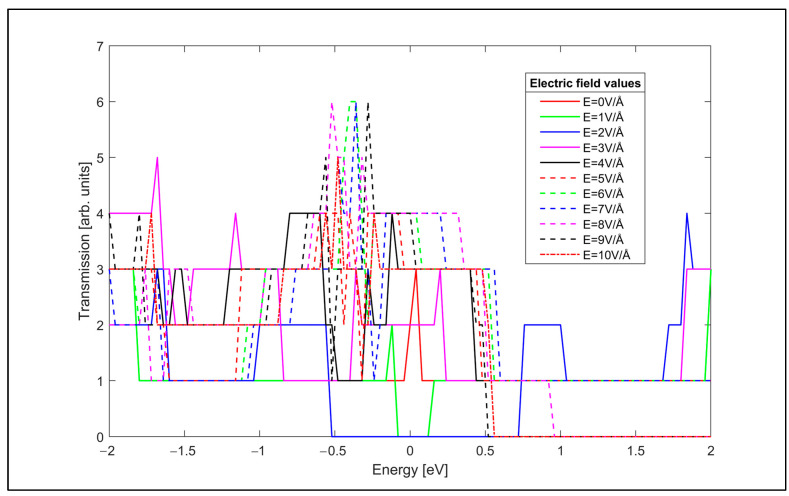
Electrical transmission spectrum of the zigzag GNR sample for various transverse electric field values.

The transverse electric field modulation of the optical characteristics of the considered zigzag and armchair GNR samples explained in the previous subsection indicates that the transverse electric field affects the band structure and the density of states of the GNRs, therefore it is important to study the electrical transmission characteristics of the zigzag and armchair GNR samples under the effect of the transverse electric field. Therefore, the electrical transmission characteristics of the zigzag and armchair GNR samples are investigated as the next step. DFT in conjunction with NEGF formalism is utilized to obtain the electrical transmission spectra of the GNR samples. The transmission spectrum of the zigzag GNR sample for various values of the applied transverse electric field are plotted in Figure 10.

**Figure 11:**
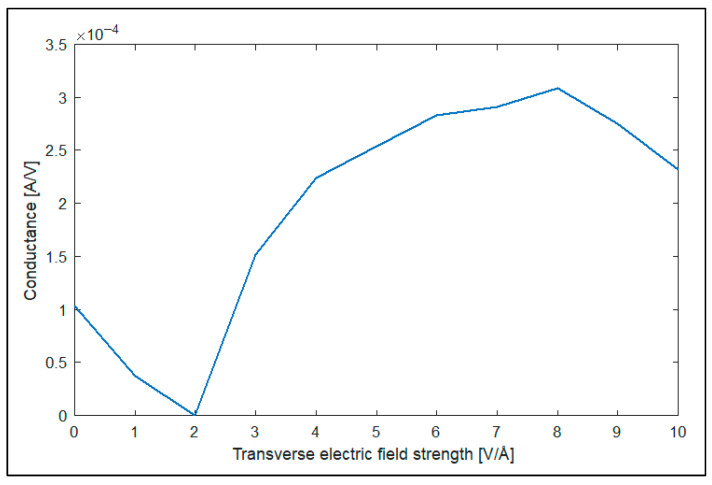
Variation of the conductance of the zigzag GNR sample dependent on the applied transverse electric field value.

As can be observed from Figure 10, the applied transverse electric field strongly affects the electrical transmission spectrum of the zigzag GNR sample, as in the optical case. The conductance of the zigzag GNR sample is also computed using the obtained transmission spectra using Landauer’s formula of Equation (6) and plotted dependent on the applied transverse electric field in Figure 11.

**Figure 12:**
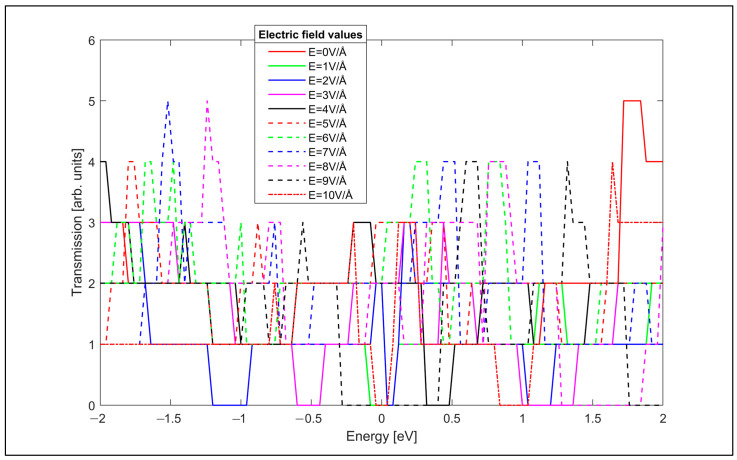
Electrical transmission spectrum of the armchair GNR sample for various transverse electric field values.

The conductance characteristics of Figure 11 show that the transverse electric field can be used to control the conductance of the zigzag GNR sample. For example, the conductance of the zigzag GNR decreases down to G = 3.2 × 10^−7^ A/V for the transverse electric field value of E = 2 V/Å. The electrical transmission spectrum of the armchair GNR sample is also given in Figure 12.

**Figure 13:**
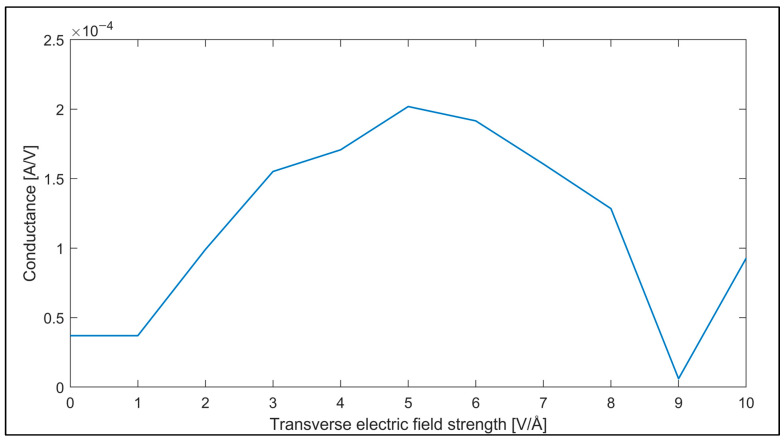
Variation of the conductance of the armchair GNR sample dependent on the applied transverse electric field value.

The electrical transmission spectra shown in Figure 12 indicate that the electrical transmission of the armchair GNR can also be controlled by changing the value of the applied transverse electric field. The conductance of the armchair GNR sample is also computed using the transmission spectra in the Landauer formula and plotted dependent on the applied transverse electric field in Figure 13. 

The conductance of the armchair GNR sample can also be adjusted by the strength of the applied transverse electric field, as in the zigzag GNR case. The conductance of the armchair GNR sample can be varied between G = 2.01 × 10^−4^ A/V and G = 6.01 × 10^−4^ A/V by adjusting the transverse electric field strength.

Both the optical and the electrical transmission analyses of the investigated zigzag and armchair GNR show that the transverse electric field can be utilized to change the optical and electrical transmission properties of these GNRs.

## 4. Conclusions

In this work, the optical and electrical transmission characteristics of zigzag and armchair GNRs under a transverse electric field are investigated using ab initio analyses. Firstly, DFT combined with Kubo–Greenwood formalism is utilized to obtain the susceptibility tensors, while DFT in conjunction with NEGF is used for obtaining the electrical transmission characteristics. As the second step, the electrical transmission behaviors of the zigzag and armchair GNR samples are investigated. The electrical transmission spectra of the GNRs are given and then the conductance of the GNR is computed via Landauer’s approach. The contributions of this study to the literature are as follows: (i) it is shown that the optical properties (complex permittivity, refractive index and reflectivity) of zigzag and armchair GNRs can be varied dependent on the transverse electric field; for example, the refractive index can be tuned in the range of n = [0.3, 9.9] and n = [0.4, 11.7] for zigzag and armchair GNR samples, respectively, for the transverse electric field strengths in the range of E = [0 V/Å, 10 V/Å]; (ii) it is found that the conductance of zigzag and armchair GNRs can be varied in the ranges of G = [0.3 μA/V, 308 μA/V] and G = [6 μA/V, 200 μA/V], respectively, while the transverse electric field value is tuned in the range of E = [0 V/Å, 10 V/Å]. These findings highlight the significant potential of GNRs for tunable optoelectronic applications. The ability to control the optical and electrical properties of GNRs via an external electric field can lead to the development of advanced devices with tailored functionalities. Future research could further explore the effects of different field orientations, temperatures, and doping levels to enhance the understanding and application of GNRs in nanoscale electronics and photonics. The demonstrated tunability in both optical and electrical domains underline the versatility and promise of GNRs as key materials in the next generation of nanoelectronics and optoelectronic devices.

## Data Availability

The data is made available as Supporting Information.

## Supplementary Materials

The following information can be downloaded at: https://www.mdpi.com/article/10.3390/nano14171446/s1: Optimized geometries, permittivity, refractive index, reflectivity and electrical transmission spectrum data.
